# The BPA-substitute bisphenol S alters the transcription of genes related to endocrine, stress response and biotransformation pathways in the aquatic midge *Chironomus riparius* (Diptera, Chironomidae)

**DOI:** 10.1371/journal.pone.0193387

**Published:** 2018-02-21

**Authors:** Óscar Herrero, Mónica Aquilino, Paloma Sánchez-Argüello, Rosario Planelló

**Affiliations:** 1 Grupo de Biología y Toxicología Ambiental, Facultad de Ciencias, Universidad Nacional de Educación a Distancia, UNED, Madrid, Spain; 2 Laboratorio de Ecotoxicología, Departamento de Medio Ambiente, Instituto Nacional de Investigación y Tecnología Agraria y Alimentaria, INIA, Madrid, Spain; University of Missouri Columbia, UNITED STATES

## Abstract

Bisphenol S (BPS) is an industrial alternative to the endocrine disruptor bisphenol A (BPA), and can be found in many products labeled “BPA-free”. Its use has grown in recent years, and presently it is considered a ubiquitous emerging pollutant. To date there is a lack of information on the effects of BPS on invertebrates, although they represent more than 95% of known species in the animal kingdom and are crucial for the structure and proper function of ecosystems. In this study, real-time RT-PCR was used to determine the early detrimental effects of BPS on the transcriptional rate of genes in the model species *Chironomus riparius*, specifically those related to the ecdysone pathway (*EcR*, *ERR*, *E74*, *Vtg*, *cyp18a1*) crucial for insect development and metamorphosis, stress and biotransformation mechanisms (*hsp70*, *hsp40*, *cyp4g*, *GPx*, *GSTd3*) that regulate adaptive responses and determine survival, and ribosome biogenesis (*its2*, *rpL4*, *rpL13*) which is essential for protein synthesis and homeostasis. While 24-hour exposure to 0.5, 5, 50, and 500 μg/L BPS had no effect on larval survival, almost all the studied genes were upregulated following a non-monotonic dose-response curve. Genes with the greatest increases in transcriptional activity (fold change relative to control) were *EcR* (3.8), *ERR* (2), *E74* (2.4), *cyp18a1* (2.5), *hsp70* (1.7), *hsp40* (2.5), *cyp4g* (6.4), *GPx* (1.8), and *GST* (2.1), while others including *Vtg*, *GAPDH*, and selected ribosomal genes remained stable. We also measured the transcriptional activity of these genes 24 hours after BPS withdrawal and a general downregulation compared to controls was observed, though not significant in most cases. Our findings showed that BPS exposure altered the transcriptional profile of these genes, which may have consequences for the hormone system and several metabolic pathways. Although further research is needed to elucidate its mode of action, these results raise new concerns about the safety of BPA alternatives.

## Introduction

Evidence demonstrated in recent years about the health hazards of bisphenol A (BPA)—used in plastics, receipt paper, food packaging, and other materials—has prompted industries to remove this xenobiotic from their products. Risks are based primarily on the ability of BPA to act as an endocrine-disrupting chemical (EDC) [1], its ubiquitous presence in the environment [2], and the potential hazards arising from continuous exposure of animals and humans [3]. Thus, manufacturers are substituting BPA with alternative substances, such as other bisphenols, in an attempt to circumvent international restrictions.

Bisphenol S (BPS) is a BPA analog used in a variety of industrial applications such as wash fastening agents in cleaning products, electroplating solvent, and a constituent of phenolic resin [4]. It can also be found in canned soft drinks, canned foodstuffs and thermal receipt papers, including products marked as “BPA-free” [5,6]. Because of its lower estrogenic activity and its greater stability against heating and sunlight, BPS is considered a safer alternative to BPA [7]. Although some BPA-like effects are hypothesized because of their similar chemical structures, BPS is presently unregulated and can be used without restriction, which has led to a sharp increase in its production and use [8]. Consequently, BPS has been detected in dust, water, sediment, sewage sludge and effluent samples [9,10], and in human urine and blood serum [11], at concentrations that are generally lower than BPA, but of the same order of magnitude [12].

While our understanding of BPS toxicity is still limited, available data suggests that BPS may not be a completely benign substitute for BPA. It has been reported that BPS altered basal testosterone secretion by mouse and human fetal testes [13]. In rodents, BPS decreased body weight [14], induced oxidative stress and morphological alterations in the male reproductive system [15], and affected transcription of genes related to the dopamine-serotonin systems [16]. In zebrafish, BPS altered the embryonic nervous and endocrine systems [17], and induced developmental and reproductive abnormalities [18]. *In vitro* studies have found that BPS induces morphological and biochemical alterations in different types of human blood cells, alters the meiotic cycle of porcine oocytes, and has weak estrogenic activity [19–21]. Different cell lines have been used to demonstrate that BPS has estrogenic potential, disrupts estradiol-induced cell signaling, modifies gene expression, induces lipid accumulation and differentiation, and alters steroidogenesis and inhibits hormone production [22–24].

Despite the fact that invertebrates represent more than 95% of known species in the animal kingdom and are extremely important regarding ecosystem structure and function, to date there is little information on the effects of BPS in these organisms. Studies with the nematode *Caenorhabditis elegans* described severe reproductive defects [25] and behavioral changes [26] caused by exposure to 125–500 μM or 0.1–10 μM BPS, respectively. The lack of information is a major concern, given that BPS is a ubiquitous pollutant that can be found in almost any environmental compartment, is able to interact with numerous invertebrate species, and thus may disrupt trophic chains and alter the natural balance of ecosystems.

Since water usually constitutes the main vehicle for dispersion of anthropogenic pollutants, aquatic ecosystems are especially sensitive to their presence. Aquatic insects are among the most important components of a freshwater ecosystem’s biota, and the family Chironomidae (Diptera) is predominant in abundance and diversity. In this regard, *Chironomus* midges have routinely been used to assess water quality by means of classical ecotoxicological endpoints, such as survival, growth, immobilization, development, and reproduction, among others [27].

Complementary to those traditional methods, molecular biomarkers in *Chironomus riparius* including gene transcription and enzyme activity have been demonstrated in recent years to be effective for the early detection of chemical toxicity, constituting an important time- and cost-effective alternative for larger-scale evaluations. Thus, *C*. *riparius* larvae have been used to assess transcriptional alterations induced by a variety of pollutants under different stress conditions, and to test their effects on the activity of enzymes involved in phase I and phase II detox pathways. There have been many effects related to endocrine disruption, specifically the ecdysone hormone, caused by BPA, phthalates, UV filters, biocides, metals, nonylphenol (NP), and nanoparticles (e.g. [28–31]). Furthermore, stage- and sex-dependent transcriptional modulation of several ecdysone-related genes has been reported throughout *C*. *riparius* development [32], and also some endocrine responses in natural populations exposed to complex mixtures of chemicals [33]. These alterations on the ecdysone pathway could ultimately affect other ecdysteroids such as 20-hydroxyecdysone (20E), the active form of the ecdysone hormone, which controls the ecdysis (molting) and metamorphosis of arthropods.

The aim of the present study was to determine for the first time the transcriptional alterations induced in *C*. *riparius* larvae by exposure to environmentally-relevant concentrations of BPS. The pathways studied include the ecdysone hormonal pathway, which plays a central role during insect development and metamorphosis, several detoxication and stress response genes that are involved in resistance mechanisms and survival, and different ribosomal genes which are important for maintenance of protein synthesis and homeostasis.

## Materials and methods

### Test animals

*C*. *riparius* populations were maintained at the facilities of the Laboratory for Ecotoxicology of INIA following laboratory conditions described by Sánchez *et al*. [34]. Briefly, larvae were reared in glass aquaria with sand sediment and spring water under static flow and gentle aeration, a photoperiod of 16/8 h of light/darkness and a temperature of 20±1°C. Commercial fish food, TetraDiscus^®^ (530 mg) was added three times a week and the rearing container covered to prevent escape of midges. Emerging adults were left to permit mating and oviposition. After removal of adults by aspiration, egg ropes were collected and used to initiate the next generation.

### Treatments

BPS (CASRN 80-09-1; purity ≥98%; Sigma-Aldrich, USA) was dissolved in dimethyl sulfoxide (DMSO; CASRN 67-68-5; purity ≥99.9%; Sigma-Aldrich) to obtain a stock solution of 1 g/L, and stored at −20°C. Working solutions of BPS (0.5 μg/L, 5 μg/L, 50 μg/L, 500 μg/L) were prepared fresh for each experiment from the stock solution diluted in culture medium, and contained 0.05% DMSO. Experiments were carried out exclusively using early phases of fourth instar larvae, which were determined based on head capsule width. Groups of 20 larvae in glass vessels were exposed to 200 ml of each BPS solution for 24 h (24h condition). At the same time, for the recovery studies, the same conditions were maintained and after exposure, BPS solutions were removed to allow larvae to recover, maintaining them in fresh culture medium for an additional 24 h (24+24h condition). After each time point of the experiment, survival values were recorded and five larvae from each vessel were collected in 1.5 ml microtubes and frozen immediately on dry ice for gene expression studies. Samples were stored at -80°C until RNA isolation; four independent experiments were performed.

### RNA isolation and cDNA synthesis

Total RNA was extracted using TRIzol Reagent (Invitrogen, Germany), following the manufacturer’s instructions. Subsequently, RNA was treated with RNase-free DNase (Roche, Germany) and extracted with phenol:chloroform:isoamyl alcohol (Fluka, Germany) using 5PRIME Phase Lock Gel Light tubes (Quantabio, USA). Purified RNA was resuspended in diethylpyrocarbonate (DEPC) water, quantified by spectrophotometry at 260 nm using a BioPhotometer (Eppendorf, Germany), and stored at -80°C.

Aliquots containing 5 μg of isolated RNA were reverse-transcribed in a C1000 Thermal Cycler (Bio-Rad, USA) using iScript Reverse Transcription Supermix for RT-qPCR (Bio-Rad), according to the manufacturer’s protocol. The obtained cDNA was conserved at -20°C and used as the template for subsequent qPCR analyses.

### Real-time RT-PCR

Quantitative real-time RT-PCR (qRT-PCR) was performed in a CFX96 Real-Time Detection System (Bio-Rad) using the Quantimix Easy Kit (Biotools, Spain). Genes encoding actin and the 26S ribosomal subunit were used as endogenous reference controls to analyze the relative transcriptional activity of selected target genes: *EcR*, *ERR*, *E74*, *Vtg*, *hsp70*, *hsp40*, *cyp18a1*, *GAPDH*, *cyp4g*, *GPx*, *GSTd3*, *its2*, *rpL4*, and *rpL13*. Primer sequences and efficiencies are shown in Table 1. The qRT-PCR was run using the following conditions: 30 s initial denaturation at 95°C, followed by 35 cycles of 5 s denaturation at 95°C, 15 s annealing at 58°C and 10 s elongation at 65°C. To verify the accuracy of each amplicon, a melting curve analysis was performed after amplification. CFX Manager 3.1 software (Bio-Rad) was used to determine total mRNA levels by normalizing the expression (2^−ΔCq^) of the target genes against the two endogenous reference genes. Each sample was run in duplicate wells, and three independent replicates were performed for each experimental condition.

**Table 1:** Primers used for quantitative real-time RT-PCR.

Gene	Description	Primer (5’→3’)	Efficiency(%)
*26S*	26S ribosomal ribonucleic acid	(**F**) TTCGCGACCTCAACTCATGT	96.6
		(**R**) CCGCATTCAAGCTGGACTTA	
*actin*	Beta-actin	(**F**) GATGAAGATCCTCACCGAACG	96.1
		(**R**) CGGAAACGTTCATTACCG	
*cyp18a1*	Cytochrome P450 18a1	(**F**) GTTTCACTCGAGACGATCCA	104.5
		(**R**) TTTAGCGGCTTGAAATGTTG	
*cyp4g*	Cytochrome P450 family 4 subfamily G	(**F**) GACATTGATGAGAATGATGTTGGTG	101.7
		(**R**) TAAGTGGAACTGGTGGGTACAT	
*E74*	Early ecdysone-inducible gene	(**F**) TCTTACTGAAACTTCTTCAAG	105.4
		(**R**) GCTTTGAGACAGCTTTGGAAT	
*EcR*	Ecdysone receptor	(**F**) TCTTCTCACGGCCATCGTCA	103.2
		(**R**) GCTGCATCTTGTTTCGCCAC	
*ERR*	Estrogen-related receptor	(**F**) CTCAGCAAGTAAGGAGGAG	99.4
		(**R**) CGTCTAATAATGTGATCGG	
*GAPDH*	Glyceraldehyde 3-phosphate dehydrogenase	(**F**) GGTATTTCATTGAATGATCACTTTG	103.9
		(**R**) TAATCCTTGGATTGCATGTACTTG	
*GPx*	Glutathione peroxidase	(**F**) AAGTGTGGTTACACAGCTAAGCATT	100.4
		(**R**) GATATCCAAATTGATTACACGGAAA	
*GSTd3*	Glutathione S-transferase D3	(**F**) TGGTTGAAACGAGAGCACCA	109.3
		(**R**) TCGGATATAGAGTGCCAGCATCG	
*hsp40*	40 kDa heat-shock protein	(**F**) TACGTGACGCTAGAGGAAA	108.1
		(**R**) TTCCAGCCCGGCTT	
*hsp70*	70 kDa heat-shock protein	(**F**) ACTTGAACCAGTTGAGCGT	102.8
		(**R**) TTGCCACAGAAGAAATCTTG	
*its2*	Internal transcribed spacer 2	(**F**) TCATCAAAGCCGTTGTCT	97.2
		(**R**) AATCGAATTGCAAACACC	
*rpL4*	Ribosomal protein L4	(**F**) AACGCTTCAGAGCTGGACGTGG	110.7
		(**R**) ATTCATCTTGTGTACGCTCATTG	
*rpL13*	Ribosomal protein L13	(**F**) AAGCTGCTTTCCCAAGAC	103.3
		(**R**) TTGGCATAATTGGTCCAG	
*Vtg*	Vitellogenin	(**F**) GATTGTTCCATGTGCAG	96.8
		(**R**) TTTGAGTATGGTGGAGAATC	

### Statistical analysis

Statistical analyses were performed using SPSS Statistics 22 software (IBM, USA). Normality and homoscedasticity of data were assessed with Shapiro-Wilk’s test and Levene’s test, respectively. Normalized levels of transcripts were analyzed with ANOVA, followed by Games Howell’s or Bonferroni’s post hoc tests, when appropriate. The Kruskal-Wallis’ test was used when data were not homogeneous or normally distributed, and the differences between pairs were established using Mann-Whitney’s tests. A probability (*P*) value ≤0.05 was used as a cutoff for statistical significance (*) of differences between treatments and control samples.

## Results

After exposure of fourth instar *C*. *riparius* larvae to 0.5, 5, 50 and 500 μg/L BPS, there was no significant effect on survival rate, neither after 24-hour continuous exposure to BPS nor in 48-hour experiments (24-hour exposure and 24 h of recovery in fresh culture medium). Survival percentages are presented in Table 2.

**Table 2:** Effect of BPS on the survival rate (%) of *C*. *riparius* larvae (n = 80) (mean ± SD).

	BPS concentration [μg/L]				
	Control	0.5	5	50	500
24h	100 ± 0.00	100 ± 0.00	96.67 ± 2.89	100 ± 0.00	95 ± 5.00
24+24h	91.67 ± 8.33	100 ± 0.00	88.64 ± 12.65	94.44 ± 4.81	88.89 ± 19.25

**Fig 1:**
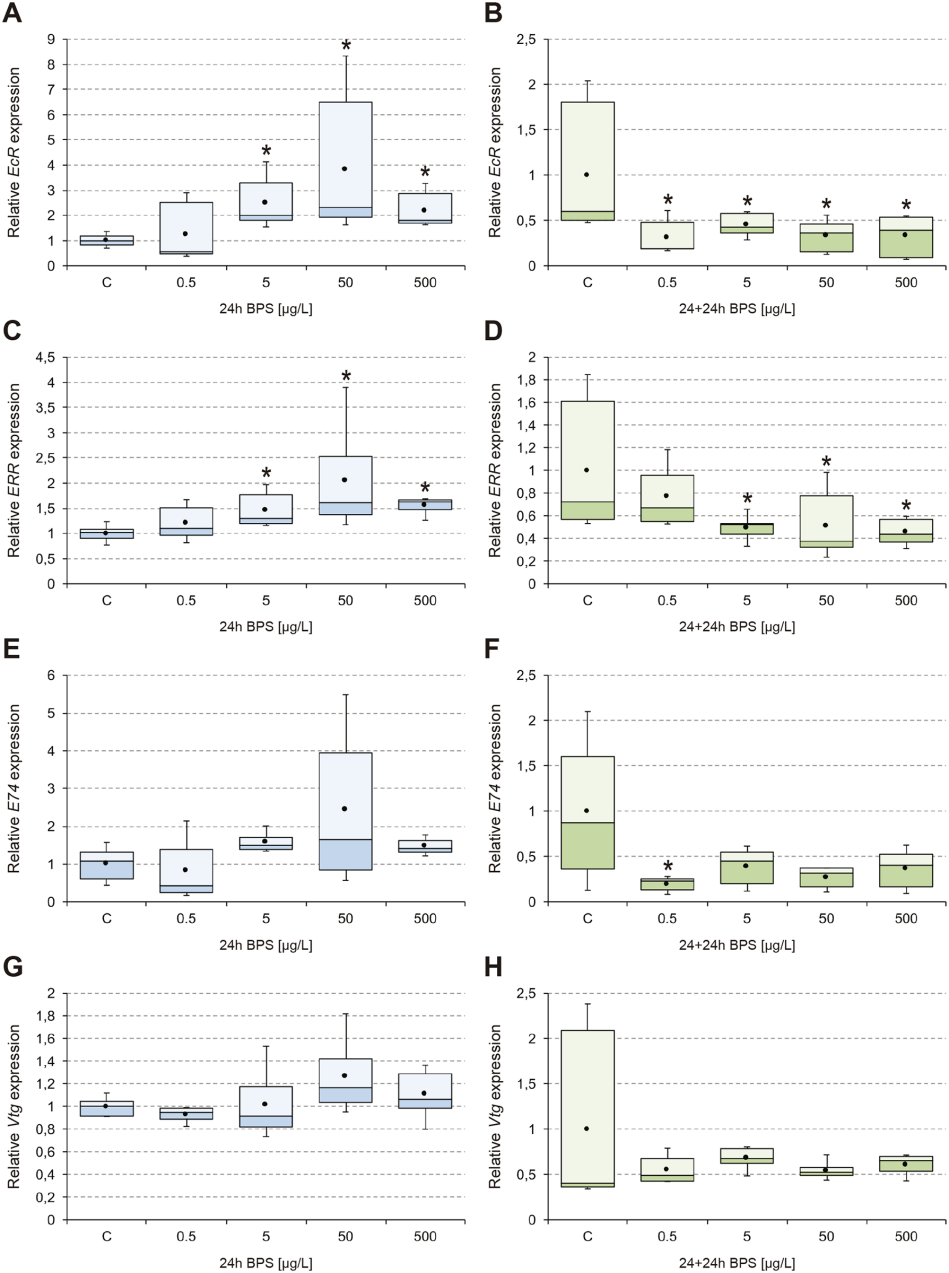
Transcriptional activity of *EcR*, *ERR*, *E74*, and *Vtg*. Box-and-whisker plots represent the expression patterns of *EcR* (A-B), *ERR* (C-D), *E74* (E-F), and *Vtg* (G-H), measured by real-time RT-PCR. *C*. *riparius* fourth instar larvae were exposed to 0.5–500 μg/L BPS for 24 h (24h; left column; blue series), and maintained for an additional 24 h in fresh culture medium after BPS withdrawal (24+24h; right column; green series). Four independent experiments were performed and RNA was extracted from 5 larvae for each experimental condition (n = 20). Results were normalized to control values. Box and whiskers represent the 25–75 percentile and the minimum/maximum measured values; the mean is represented by a dot; the horizontal line separating the lower (dark) and the upper (light) area represents the median. Asterisks (*) indicate significant differences (p ≤ 0.05) with respect to control values.

**Fig 2:**
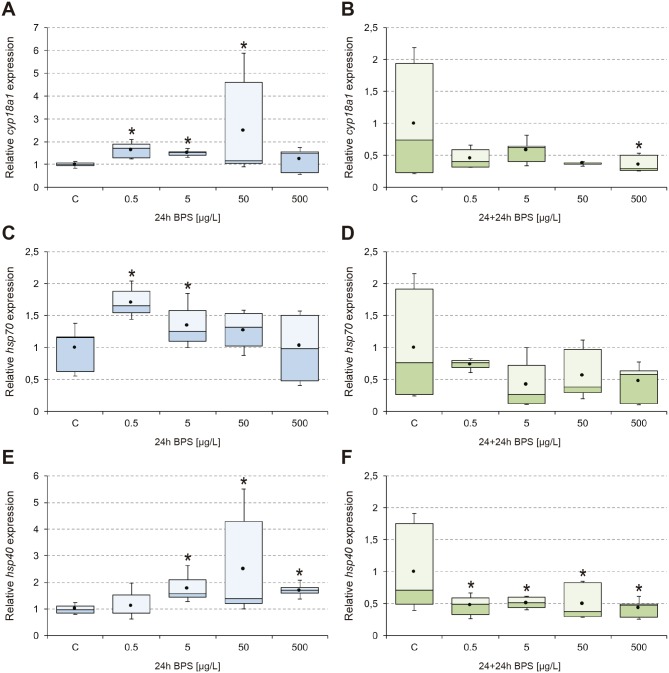
Transcriptional activity of *cyp18a1*, *hsp70*, and *hsp40*. Box-and-whisker plots represent the expression patterns of *cyp18a1* (A-B), *hsp70* (C-D), and *hsp40* (E-F), measured by real-time RT-PCR. *C*. *riparius* fourth instar larvae were exposed to 0.5–500 μg/L BPS for 24 h (24h; left column; blue series), and maintained for an additional 24 h in fresh culture medium after BPS withdrawal (24+24h; right column; green series). Four independent experiments were performed and RNA was extracted from 5 larvae for each experimental condition (n = 20). Results were normalized to control values. Box and whiskers represent the 25–75 percentile and the minimum/maximum measured values; the mean is represented by a dot; the horizontal line separating the lower (dark) and the upper (light) area represents the median. Asterisks (*) indicate significant differences (p ≤ 0.05) with respect to control values.

**Fig 3:**
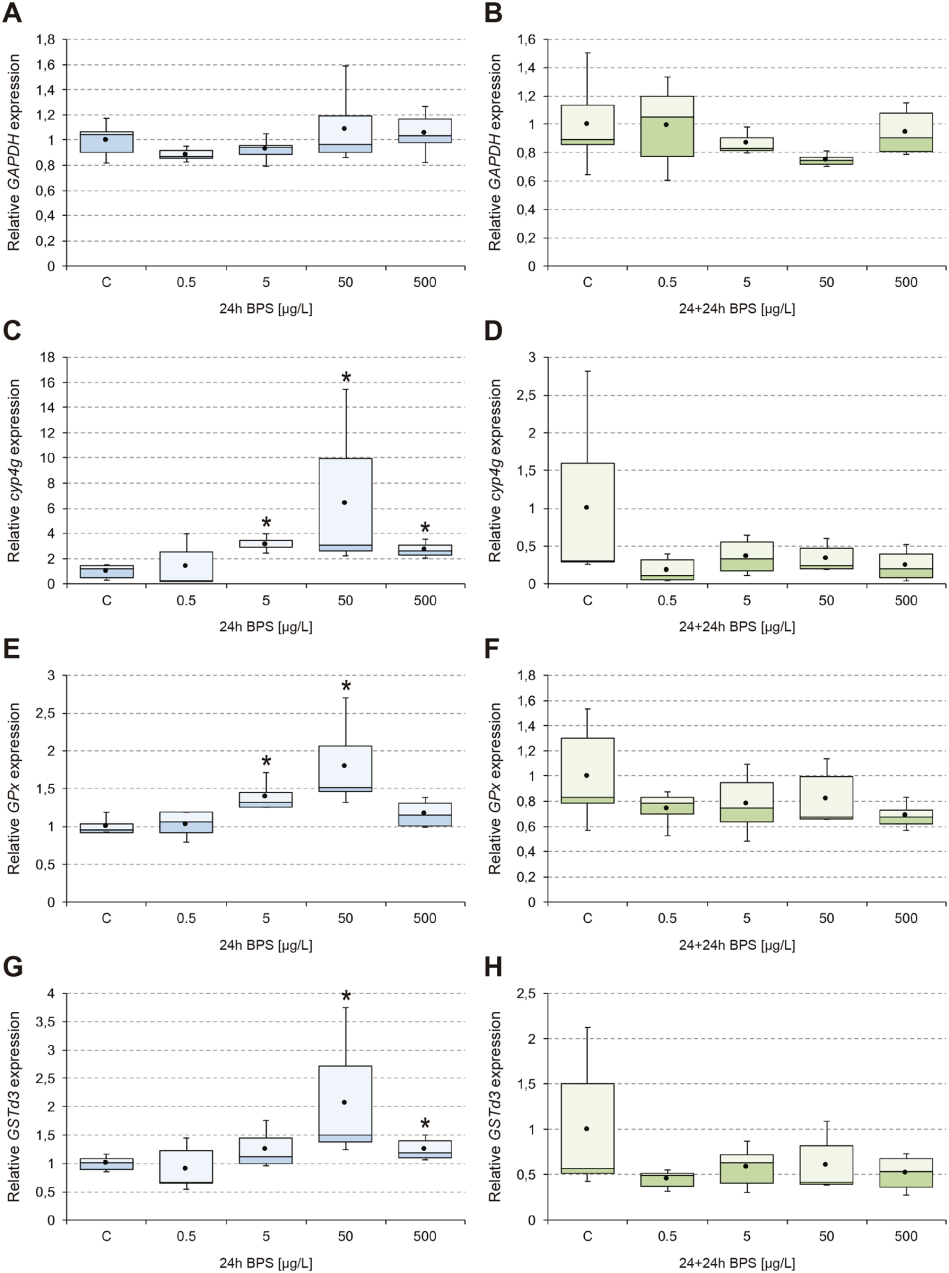
Transcriptional activity of *GAPDH*, *cyp4g*, *GPx*, and *GSTd3*. Box-and-whisker plots represent the expression patterns of *GAPDH* (A-B), *cyp4g* (C-D), *GPx* (E-F), and *GSTd3* (G-H) measured by real-time RT-PCR. *C*. *riparius* fourth instar larvae were exposed to 0.5–500 μg/L BPS for 24 h (24h; left column; blue series), and maintained for an additional 24 h in fresh culture medium after BPS withdrawal (24+24h; right column; green series). Four independent experiments were performed and RNA was extracted from 5 larvae for each experimental condition (n = 20). Results were normalized to control values. Box and whiskers represent the 25–75 percentile and the minimum/maximum measured values; the mean is represented by a dot; the horizontal line separating the lower (dark) and the upper (light) area represents the median. Asterisks (*) indicate significant differences (p ≤ 0.05) with respect to control values.

**Fig 4:**
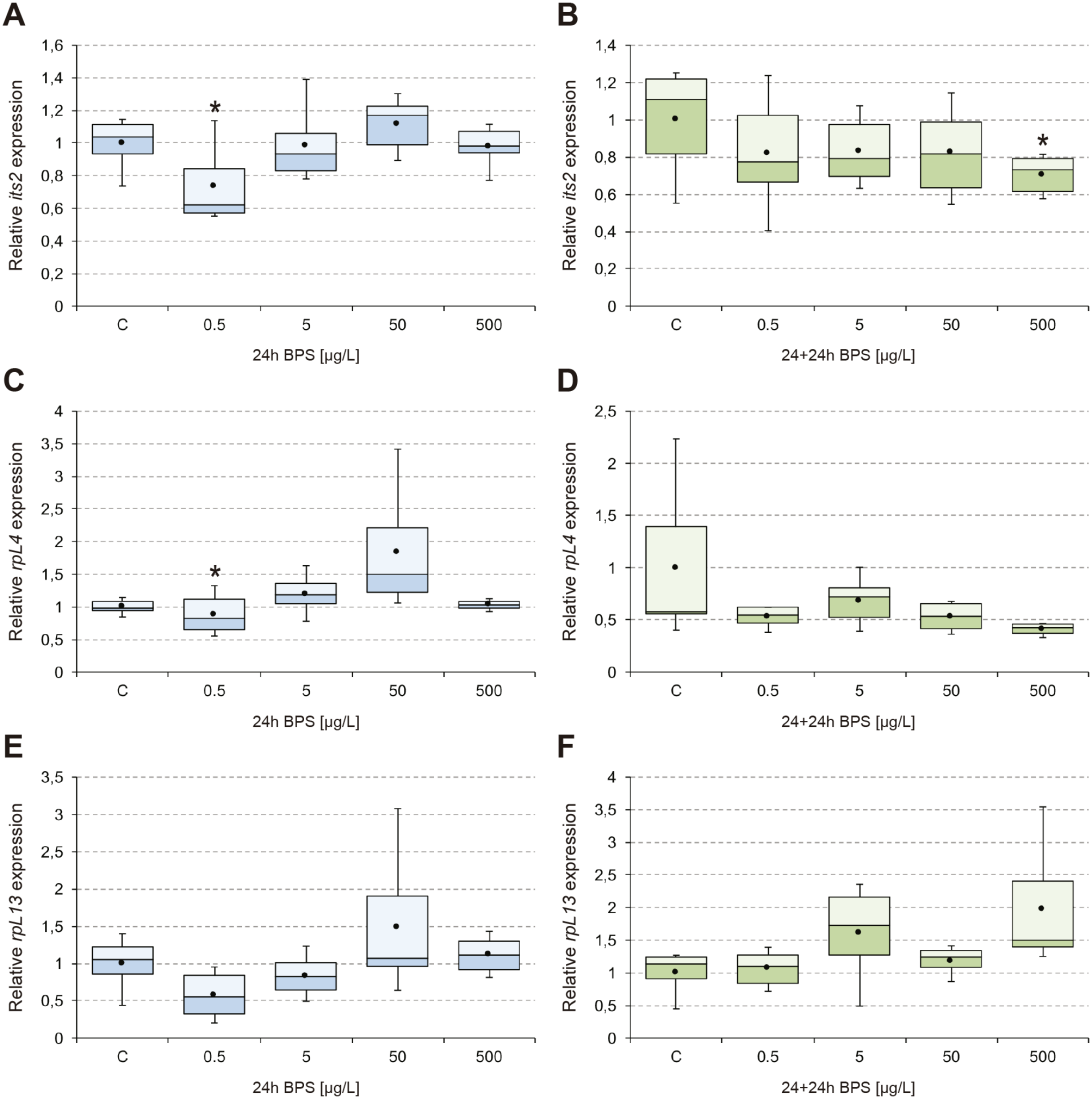
Transcriptional activity of *its2*, *rpL4*, and *rpL13*. Box-and-whisker plots represent the expression patterns of *its2* (A-B), *rpL4* (C-D), and *rpL13* (E-F), measured by real-time RT-PCR. *C*. *riparius* fourth instar larvae were exposed to 0.5–500 μg/L BPS for 24 h (24h; left column; blue series), and maintained for an additional 24 h in fresh culture medium after BPS withdrawal (24+24h; right column; green series). Four independent experiments were performed and RNA was extracted from 5 larvae for each experimental condition (n = 20). Results were normalized to control values. Box and whiskers represent the 25–75 percentile and the minimum/maximum measured values; the mean is represented by a dot; the horizontal line separating the lower (dark) and the upper (light) area represents the median. Asterisks (*) indicate significant differences (p ≤ 0.05) with respect to control values.

The results obtained from analysis of transcriptional activity of *EcR*, *ERR*, *E74*, and *Vtg* (Fig 1), *cyp18a1*, *hsp70*, and *hsp40* (Fig 2), *GAPDH*, *cyp4g*, *GPx*, and *GSTd3* (Fig 3), *its2*, *rpL4*, and *rpL13* (Fig 4) are shown. In general, 24-hour exposure to increasing doses of BPS led to an inverted U-shaped overexpression curve in which the maximum transcriptional activity appeared at the concentration of 50 μg/L, while withdrawal of the compound led to a generalized downregulation 24 h later. This peak transcription at 50 μg/L was statistically significant compared to 5 μg/L and 500 μg/L BPS, for the endocrine and detoxication genes analyzed.

Genes encoding nuclear receptors were significantly overexpressed after 24 h exposure to 5 μg/L BPS or greater, with maximum levels reached at 50 μg/L BPS, with a mean increase in transcriptional activity for *EcR* and *ERR* of 3.8-fold and 2-fold, respectively (Fig 1A and 1C). Both genes were significantly repressed in almost all conditions after BPS withdrawal, falling to values 70% lower than controls (Fig 1B and 1D). Similarly, the expression profile of *E74* had a peak after exposure to 50 μg/L BPS (2.4-fold) (Fig 1E) and a general downregulation after 24 h recovery (up to 80% less than control) (Fig 1F). However, in this case downregulation was not significant except at the lowest BPS concentration tested. More subtle changes were observed for *Vtg*, which did not exhibit statistical significance in any of the experimental conditions (Fig 1G and 1H).

Transcriptional activity of *cyp18a1* was significantly upregulated from 0.5 μg/L BPS and reached its maximum (2.5-fold increase) at 50 μg/L BPS (Fig 2A). Withdrawal of BPS led to a general reduction of activity (50% less than control), which was statistically significant at the highest dose (500 μg/L) (Fig 2B). The two genes encoding heat shock proteins had increased expression levels in the presence of BPS, although they showed a differential response. While *hsp40* showed significant differences above 5 μg/L BPS (Fig 2E), similar to other genes in this study, *hsp70* showed a negative, linear dose-response, with significant differences only at the two lowest doses (Fig 2C). Both genes were downregulated after BPS withdrawal (up to 60% less) (Fig 2D and 2E), with *hsp40* significantly lower than the control at all concentrations (Fig 2E).

*GAPDH* remained unaltered under all the experimental conditions (Fig 3A and 3B). However, genes involved in biotransformation activities (*cyp4g*, *GPx*, and *GST*) showed similar responses to those observed for *EcR*, *ERR*, *cyp18a1*, and *hsp40*, with significant overexpression after 24 h exposure to 5 μg/L BPS and a maximum value at 50 μg/L (6.4-fold, 1.8-fold, and 2.1-fold, respectively) (Fig 3C, 3E and 3G). All these genes were also repressed after BPS withdrawal, although our results did not have significant differences (Fig 3D, 3F and 3H).

Ribosomal genes remained fairly stable, although some effects were observed. The lowest BPS dose (0.5 μg/L) caused a drop in transcription, significant for *its2* and *rpL4* (about 25% below controls) (Fig 4A and 4C). On the contrary, and similar to that observed for other genes (although without statistical significance), maximum transcription was observed after exposure to 50 μg/L BPS (Fig 4A, 4C and 4E). After recovery following BPS removal, both *its2* and *rpL4* were repressed (Fig 4B and 4D), while *rpL13* showed a rise in its transcription rate (up to 2-fold) that was not significant due to data variability (Fig 4F).

## Discussion

Many works in recent years have revealed the dangerous nature of BPA, not only regarding hormonal alteration and its implication in reproduction and development, but also other health disorders such as breast cancer, obesity, type-2 diabetes, oxidative stress, neurodevelopment, asthma, and cardiovascular diseases [1]. With this evidence, and thanks to different social and legal pressures, industries have gradually replaced BPA with alternatives including structural analogs such as bisphenols AF, AP, B, E, F, P, S, or Z. This replacement has been carried out without strict adherence to the “precautionary principle”, which should be used when preliminary scientific evidence indicates that there are reasonable concerns about potentially dangerous effects on the environment or human, animal or plant health [35]. Thus, as more scientific studies show adverse effects of these chemical alternatives [8,13,36], it may be just as risky to buy a product labeled "BPA-free" as opting for those containing BPA.

The objective of the present work was to shed light on the possible adverse environmental effects of BPS, a BPA analogue commonly used in "BPA-free" products. Our experiments were carried out with aquatic larvae of *C*. *riparius* midges, a very relevant species in aquatic ecosystems used in studies on ecotoxicity of water and sediments, and especially suitable for risk assessments of potential endocrine-disrupting properties of chemicals [37,38]. For this purpose, we selected BPS concentrations ranging from 0.5 to 500 μg/L, where the lowest doses are of the same order of magnitude as those detected in natural environments, wastewater, and the urine of exposed populations. Some mean maximum concentrations reported in aquatic environments are 0.16 and 1.6 μg/L for Taihu Lake (China) [39,40], and 7.2 μg/L for Adyar River (India) [41]. Sun *et al*. [10] reported values ranging from 0.183–0.746 μg/L (influent) in a wastewater treatment plant in China. Urine samples from Japan, USA, and China contained 0.933, 0.304, and 0.223 μg/ml BPS, respectively [42]. BPS is persistent in sediments and more resistant to biodegradation than BPA in aquatic environments, which may lead to its accumulation in the biota, and thus a greater exposure for species of the affected ecosystems. In addition, the concentrations in this study were in the range of those tested in different studies with other species, especially zebrafish (up to 1000 μg/L) (e.g. [18,43,44]).

Exposure to BPS had no significant effect on larval survival, similar to previous 24-hour studies with BPA in which *C*. *riparius* larvae did not show mortality even at doses up to 3,000 μg/L [31,45]. This allowed us to analyze the effects under sublethal conditions, which is particularly useful to assess cellular, molecular, or biochemical alterations, and may also better estimate responses of benthic communities to contaminants.

The general transcriptional profile of genes found to be affected after 24-hour exposure to BPS, especially those involved in endocrine and biotransformation pathways, followed a non-monotonic dose-response curve (NMDRC), consisting of a biphasic, inverted U-shape. NMDRCs and the presence of low-dose effects are characteristics of EDCs, and are especially problematic for assessing potential impacts of exposure [1,46] given that they do not fit linear (positive or negative) relationships typically observed in toxicological assessments and do not follow the dogma of “the dose makes the poison”. It is noteworthy that these curves may also present problems for extrapolation of results from lower or higher doses [46], which must be considered in future studies on the effects of BPS. There is an increasing body of evidence that supports gene expression profiles at low doses differ from those at high doses, indicating that hormesis (both monotonic and non-monotonic dose-response) is not limited to a simple adaptive response. Furthermore, adverse stimulatory effects produced by EDCs observed as inverted U-shape dose-response challenge the current methods for risk assessment [47].

BPS led to an increase in the transcriptional activity of the gene encoding the ecdysone receptor (*EcR*), possibly disrupting the genetic cascade of this hormonal pathway (Fig 1A). It has been described that this rise in the transcriptional activity of *EcR* triggers activation of a conserved hierarchical cascade of transcription factors encoded by early ecdysone-responsive genes, including *E74* [48]. In insects, this coordinated response is crucial in developmental processes that regulate molting and metamorphosis ([32] and references therein). Our results confirmed that such *EcR* upregulation was concurrent with an increase in transcriptional activity of *E74* (Fig 1E). Removal of BPS turned these agonistic responses into antagonistic effects, manifested as repression of these genes, where inactivation of *EcR* (Fig 1B) may have conditioned the transcriptional responses of other genes involved in the ecdysone regulatory cascade (Fig 1F). This would be consistent with the harmonized response described during development of *C*. *riparius* and other insects [32]. Although previous studies have reported a lower hormonal activity of BPS compared to BPA [8,19], it is noteworthy that the endocrine-disrupting effect identified in our study was markedly higher than that detected in *C*. *riparius* larvae exposed to BPA [31]. In that study, a much higher dose of BPA (3000 μg/L) was necessary to produce half the effect on *EcR* (near 2-fold upregulation) than 50 μg/L BPS (near 4-fold).

Nuclear receptors (NRs) are one of the primary targets of EDCs and comprise a superfamily of proteins that perform critical functions in the hormone system; estrogen-related receptors (ERRs) belong to the subgroup of steroid hormone receptors [49]. Like many other NRs, ERRs function as both repressors and activators of gene expression and play specific roles during development and adult function, influencing a wide range of physiological processes including those governed by classic ERs. A previous study in which *C*. *riparius* larvae were exposed to BPA under similar dose and time parameters (5, 50, 500 μg/L; 24 and 96 h) showed an equivalent upregulation of *ERR* [49], though with a positive linear dose-response relationship instead of the NMDRC induced by BPS in the current study (Fig 1C). In the present work, BPS exhibited an ability to interact with NRs other than the ecdysone receptor, which showed similar responses to *EcR* both during BPS exposure (Fig 1A and 1C) and after its removal (Fig 1B and 1D). In *Chironomus dilutus*, it has been suggested that effects on *ERR* might be due to estrogen-like compounds [50], so taken together our results indicate that BPS induces significant estrogenic activity.

Vitellogenin is an egg yolk precursor protein that is expressed in many oviparous vertebrates and invertebrates, and is a sensitive biomarker for estrogen-like responses. Regulation of *Vtg* in the fat body by ecdysteroids occurs by a conserved set of ecdysone-responsive early and early-late genes [51]. In the present study, no significant changes in expression of *Vtg* were induced by BPS (Fig 1G and 1H), while a previous study on the effects of BPA showed its significant upregulation under equivalent concentrations (5, 50, 500 μg/L), but after 96-hour exposures [52]. In zebrafish (*Danio rerio*), significant overexpression of *Vtg* has been reported after 75 days of exposure to 10 and 100 μg/L BPS [18]. Therefore, longer exposures of *Chironomus* larvae to BPS might be necessary to trigger a transcriptional effect on *Vtg*. Other environmental EDCs have induced [52], or not [53], the expression of *Vtg* in *C*. *riparius*. Our results are consistent with the fact that 20E (or analogs) does not stimulate vitellogenin synthesis in the fat body *in vitro* although it stimulates general protein synthesis [54]. Nevertheless, further investigation using insect life-cycle tests are necessary to clearly establish the effects of BPS on vitellogenesis.

Cytochrome P450 enzymes are involved in a number of steps in ecdysteroid homeostasis in insects, thus conditioning their major developmental transitions. The *cyp18a1* gene is conserved in most arthropods and it is essential for proper development of these organisms [55]. CYP18A1 plays a role in catabolism of 20E, the active form of the ecdysone hormone, catalyzing 26-hydroxylation of ecdysteroids. *cyp18a1* has been shown to be induced by ecsdysteroid-like compounds, and a role has been suggested in the preparation for metamorphosis and molting [56]. Previous work with the EDC vinclozolin showed increased transcription of *EcR*, *E74* and *cyp18a1* in *C*. *riparius* larvae after exposure, but contrary to our study (Fig 2A and 2B), not in a coordinated way, as peaks for *EcR* did not fit with those for *E74* or *cyp18a1* [28]. Given that both ecdysone and 20E are substrates of CYP18A1 [55], our results suggest that BPS might behave as an ecdysone agonist.

Heat-shock proteins (HSPs) are a group of proteins that function to reverse or inhibit denaturation or unfolding of cellular proteins in response to a variety of environmental stressors, including high temperature. HSP70 has crucial functions in protein folding, maintenance of protein homeostasis, and enhancement of cell survival following a multitude of stresses [57]. HSP40 is a HSP70 co-chaperone that can stimulate the ATPase activity of HSP70 and regulate protein folding, unfolding, translation, translocation, and degradation. In addition, HSP70 is among the auxiliary factors necessary, at least transiently, for proper function of the ecdysone receptor and its binding to the hormone [58]. Both *hsp70* and *hsp40* are inducible genes that have been shown to modulate their transcriptional activity under exposure to different xenobiotics. Particularly in *C*. *riparius*, two previous studies have described the overexpression of *hsp70* after 24-hour exposure to high (3000 μg/L) [31] and low (8, 80, 800 μg/L) [59] BPA concentrations. While Lee *et al*. [59] described a positive linear dose-response relationship, our results in this study showed a negative relationship, with *hsp70* reaching maximum transcription after exposure to the minimum concentration of BPS tested (Fig 2C). It should be noted that *hsp70* was the only gene without a NMDRC, which may be due to the fact that HSP70 is implicated in a multitude of processes focused on maintaining cellular homeostasis [57], and thus might not only be affected by the endocrine-disrupting effects of BPS. Upregulation of *hsp40* (Fig 2E), and its subsequent repression after BPS withdrawal (Fig 2F), are in line with the effects described for the nuclear receptors, so its activity may be linked to post-transcriptional processes derived from the hormonal activity of BPS.

No significant changes were detected in the transcription of *GAPDH* (Fig 3A and 3B), which remained stable under our experimental conditions. This is consistent with the traditional use of this gene as a reference “housekeeping” gene in transcriptional studies [60], though it has been demonstrated that its expression may sometimes be altered under xenobiotics-induced stress [61,62].

BPS effects on detoxication activities in *C*. *riparius* were assessed by analyzing the transcriptional profile of several genes involved in phase I (*cyp4g*) and phase II (*GPx*, *GSTd3*) biotransformation reactions. Those reactions are evolutionarily conserved and are crucial for the maintenance of cell homeostasis, by reducing the harmful effects of reactive oxygen species (ROS) and xenobiotics [63,64]. In a recent *in vitro* study with human red blood cells, it was found that 250 μg/ml BPS increased ROS levels [21]. A previous study reported that BPS induces oxidative stress in mouse liver and renal cells [65]. Our results showed a NMDRC with maximum peaks of transcription of *cyp4g*, *GPx*, and *GST* after exposure to 50 μg/L BPS (6.4-fold, 1.8-fold, and 2.1-fold, respectively) (Fig 3C, 3E and 3G). It should also be noted that *cyp4g* was transcribed 2.7-fold above the untreated control at 500 μg/L (Fig 3C), and that under the same exposure conditions, BPA caused repression of this same gene (50% of control) in a previous *C*. *riparius* experiment [66]. In this sense, it seems clear that these chemical analogs trigger different responses in the activity of *cyp4g*. Based on the literature, it is reasonable to expect that the activation of these detox genes might lead to an adaptive response to the toxic effects of BPS, especially those related to oxidative stress.

Finally, our results demonstrated that genes related to ribosomal biogenesis (*its2*, *rpL4*, *rpL13*) remained stable (Fig 4), although a significant decrease was detected in the lowest dose analyzed (0.5 μg/L BPS) for its2 and rpL4 (Fig 4A and 4C). As they constitute the ribosome, machinery necessary for protein synthesis, they are usually constitutively expressed and therefore considered “housekeeping” genes [60]. However, different xenobiotics have a demonstrated ability to modify the transcription of these genes [61,67]. Under our experimental conditions, it seems that the ribosomal function are not a specific target of BPS toxicity.

## Conclusions

The present work shows for the first time in invertebrates that BPS activates transcription of genes encoding NRs, which may possibly interfere with ecdysone receptor function. This activation was concomitant with an increase in expression levels of other genes involved in the hormonal pathway mediated by ecdysone, thus BPS may be acting as a hormone agonist. These responses followed a NMDRC, which is particularly characteristic of EDCs. The compound was also capable of activating genes involved in the cellular stress response and in phase I/II detoxication mechanisms. Although low, environmentally-relevant concentrations of BPS (0.5–500 μg/L) did not compromise the survival of *C*. *riparius* larvae after 24 h of exposure, it is important to take into account that its biodegradation-tolerance and capacity to accumulate in the biota could lead to future scenarios of higher exposure. Although it is not possible to know the environmental implications of BPS exposure based exclusively on transcriptional results, we may consider that these alterations at the molecular level might ultimately be reflected at the organism / population / ecosystem level. Further research is needed to properly describe the mode of action of this xenobiotic in insects, though the available data suggests that it may not be a completely benign substitute for BPA.
